# Influence of weather on gobbling activity of male wild turkeys

**DOI:** 10.1002/ece3.9018

**Published:** 2022-06-17

**Authors:** Patrick H. Wightman, James A. Martin, John C. Kilgo, Emily Rushton, Bret A. Collier, Michael J. Chamberlain

**Affiliations:** ^1^ Warnell School of Forestry and Natural Resources University of Georgia Athens Georgia USA; ^2^ Southern Research Station USDA Forest Service New Ellenton South Carolina USA; ^3^ Georgia Department of Natural Resources – Wildlife Resources Division Social Circle Georgia USA; ^4^ School of Renewable Natural Resources Louisiana State University Agricultural Center Baton Rouge Louisiana USA

**Keywords:** acoustic monitoring, convolutional neural network, gobbling, *Meleagris gallopavo*, southeastern U.S., weather, wild Turkey

## Abstract

Gobbling activity of Eastern wild turkeys (*Meleagris gallopavo silvestris*; hereafter, turkeys) has been widely studied, focusing on drivers of daily variation. Weather variables are widely believed to influence gobbling activity, but results across studies are contradictory and often equivocal, leading to uncertainty in the relative contribution of weather variables to daily fluctuations in gobbling activity. Previous works relied on road‐based auditory surveys to collect gobbling data, which limits data consistency, duration, and quantity due to logistical difficulties associated with human observers and restricted sampling frames. Development of new methods using autonomous recording units (ARUs) allows researchers to collect continuous data in more locations for longer periods of time, providing the opportunity to delve into factors influencing daily gobbling activity. We used ARUs from 1 March to 31 May to detail gobbling activity across multiple study sites in the southeastern United States during 2014–2018. We used state‐space modeling to investigate the effects of weather variables on daily gobbling activity. Our findings suggest rainfall, greater wind speeds, and greater temperatures negatively affected gobbling activity, whereas increasing barometric pressure positively affected gobbling activity. Therefore, when using daily gobbling activity to make inferences relative to gobbling chronology, reproductive phenology, and hunting season frameworks, stakeholders should recognize and consider the potential influences of extended periods of inclement weather.

## Graphical abstract

Using autonomous recording units we collected gobbling activity of male wild turkeys across multiple study sites in the southeastern United States during 2014 ‐ 2018. We used state‐space modeling to investigate the effects of weather variables on daily gobbling activity and found rainfall, greater wind speeds, and greater temperatures negatively affected gobbling activity, whereas increasing barometric pressure positively affected gobbling activity. When using daily gobbling activity to make inferences relative to gobbling chronology, reproductive phenology, and hunting season frameworks stakeholders should recognize and consider the potential influences of extended periods of inclement weather.

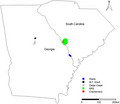

## 1 INTRODUCTION

Male birds often rely on visual and auditory courtship behaviors to portray reproductive fitness to females, attract mates, and maintain social and dominance hierarchies (Buchholz, 1997; Cornec et al., 2017; Mateos & Carranza, 1999; Omland, 1996; Williams, 1984). Frequency of courtship behaviors, such as vocalizations by males, changes in response to conspecifics along with anthropogenic and environmental influences (Berg et al., 2005; Slabbekoorn & Ripmeester, 2008; Staicer et al., 1996). Ecological theories such as the adaptive acoustic hypothesis and risk‐reward theory suggest birds adopt different vocalization strategies depending on environmental conditions to maximize the effectiveness and costs associated with calling (Lima, 2009; Luther, 2009; Orians, 1969; Zanette et al., 2006).

The wild turkey (*Meleagris gallopavo*) is a non‐migratory upland game bird indigenous to North America whose mating strategy is a form of polygamy similar to exploded lekking (Krakauer, 2008). Turkeys use a polygynous‐promiscuous mating system, where females choose males who are competing for mating opportunities via visual displaying (e.g., strutting) and auditory vocalizations (e.g., gobbling, drumming; Healy, 1992). Turkeys gobble to secure mates by attracting females, maintain territories, and compete with other males (Bailey & Rinnell, 1967; Bevill, 1973; Healy, 1992). Gobbling activity is believed to be influenced by a variety of factors, such as time of day, timing of female reproductive activities, conspecifics, hunting, weather, predation risk, age structure, and testosterone levels (Chamberlain et al., 2018; Kienzler et al., 1996; Miller et al., 1997a,b Wakefield et al., 2020; Wightman et al., 2019). Wildlife managers and agencies are interested in understanding factors influencing gobbling activity, as it is the primary determinant of hunter satisfaction and is likely linked to reproductive success (Casalena et al., 2011; Chamberlain et al., 2018; Isabelle & Reitz, 2015; Schroeder, 2014).

Historical research relied on roadside surveys to describe gobbling activity, but results from previous studies contained notable discrepancies in regard to drivers of variation in gobbling activity. For example, early studies reported both positive and negative effects of nesting phenology, weather, and hunting pressure on gobbling activity (Bevill, 1975; Kienzler et al., 1996; Lehman et al., 2005; Miller et al., 1997a, 1997b; Palumbo et al., 2019). However, no definitive relationship between any of the aforementioned variables and gobbling activity was established, likely due to a lack of uniformity in data collection, coupled with logistical difficulties in obtaining high‐quality, detailed, spatially explicit gobbling data. Furthermore, roadside surveys were generally not conducted during inclement weather and can be additionally biased by observer error, sample design, and manpower limitations (Lehman et al., 2005; Miller et al., 1997a, 1997b; Palumbo et al., 2019).

Development and use of autonomous recording units (ARU; Colbert et al., 2015; Mennill et al., 2012; Rempel et al., 2005) offer researchers the ability to thoroughly detail gobbling activity. With advancement of ARU technology, recent studies have elucidated how factors such as time of day, nesting phenology/female receptivity, and hunting influence gobbling activity using spatially and temporally robust datasets (Chamberlain et al., 2018; Wakefield et al., 2020; Wightman et al., 2019). In general, gobbling activity was highest 30 min prior to sunrise until 150‐min post‐sunrise (hereafter; daily gobbling activity) and fluctuated considerably from one morning to the next (Wightman et al., 2019). Additional work has indicated that female nesting phenology was positively related to gobbling activity, with the onset of reproductive activities resulting in an initial peak of gobbling (Chamberlain et al., 2018; Wakefield et al., 2020). Furthermore, contemporary literature has noted hunting activity may have a greater negative influence on gobbling activity than the positive effect of nesting phenology (Wakefield et al., 2020; Wightman et al., 2019). However, there is no existing literature using ARUs to investigate the relative influences of weather variation on gobbling activity.

Based on previous literature investigating calling activity of wild turkeys and other avian species, we hypothesized that increased temperature, humidity, wind, and occurrence of rain would negatively impact gobbling activity (Digby et al., 2014; Gudka et al., 2019; Lengagne & Slater, 2002; Miller et al., 1997a). Likewise, we hypothesized that increases in barometric pressure would likely positively influence gobbling activity (Pellegrino et al., 2013; Wellendorf et al., 2004). Therefore, our objectives were to evaluate potential relationships between gobbling activity of male Eastern wild turkeys (*Meleagris gallopavo silvestris*) collected using ARUs and the aforementioned weather variables across multiple study sites in the southeastern United States.

## 2 METHODS

### 2.1 Study area

**FIGURE 1:**
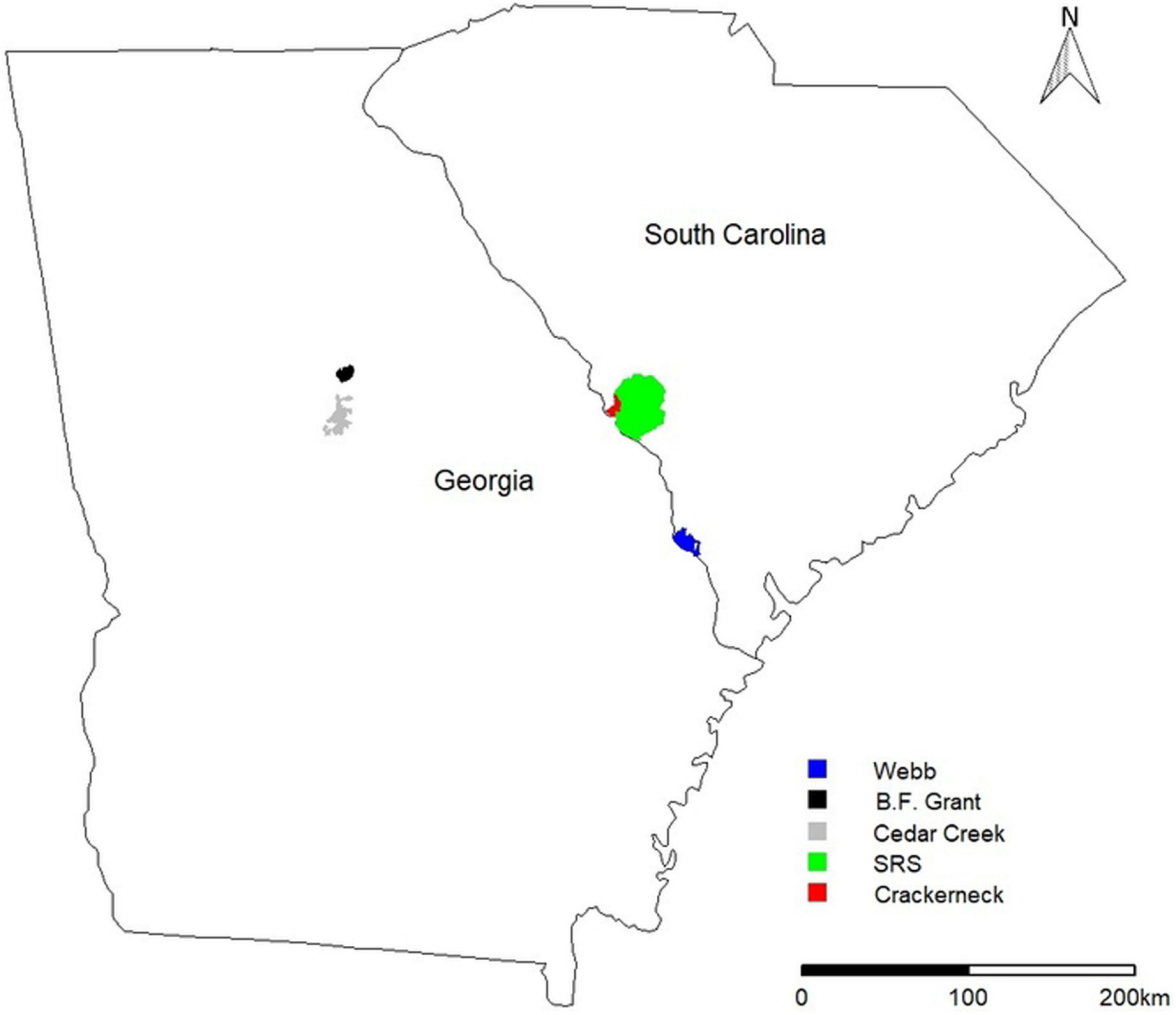
Location of Webb Wildlife Management Area Complex, Crackerneck Wildlife Management Area, the Savannah River Site in South Carolina, USA, and B.F. Grant and Cedar Creek Wildlife Management Areas in Georgia, USA

We conducted research on 5 study sites in Georgia and South Carolina, USA (Figure 1). The first site, located in Aiken County, South Carolina, was the 4400‐ha Crackerneck Wildlife Management Area (CWMA), owned by the United States Department of Energy and managed by SCDNR (South Carolina Department of Natural Resources). Landcover types on CWMA consisted of upland and bottomland hardwoods, mixed pine‐hardwoods, planted pine stands (*Pinus* spp.), and wildlife openings. Turkey hunting season opened annually on 1 April with a youth hunt on the Saturday prior, and closed on 1 May, with hunting occurring only on Fridays and Saturdays. The second site in South Carolina was the United States Department of Energy's Savannah River Site (SRS), which consisted of 78,000 ha located in Aiken and Barnwell counties. The SRS was mostly forested and consisted of upland and bottomland hardwoods, mixed pine‐hardwoods, and planted stands of longleaf (*P. palustris*) and loblolly pine (*P. taeda*). Since 1951, turkey hunting pressure on SRS was limited. Hunting was restricted to an annual 2‐day hunt during the third weekend of April for mobility‐impaired hunters that began in 2002. We collected data on CWMA and SRS during 2014–2018. For more detailed descriptions of site‐specific conditions on the South Carolina study sites, see Wightman et al. (2019).

From 2015 to 2018, we collected data on 3 contiguous Wildlife Management Areas (WMAs) known as the Webb WMA Complex in Hampton and Jasper counties in South Carolina. The Webb WMA Complex was 10,483‐ha dominated by pine forests consisting mostly of loblolly pine and longleaf pine, with hardwood stands adjacent to riparian drainages, and bottomland hardwoods and wetlands along the Savannah River. The Webb WMA Complex was actively managed for a variety of wildlife by the SCDNR. Hunting season for male turkey opened annually on 1 April with a youth hunt on the Saturday prior and ended in the first week of May, and hunting was permitted on Monday–Saturdays.

During 2017–2018, we collected data on two WMAs in the Piedmont region of Georgia, USA. Cedar Creek WMA (CCWMA) was a 16,187‐ha area located in Jasper, Jones, and Putnam counties owned by the United States Department of Agriculture Forest Service (USFS) and managed in partnership with the Georgia Department of Natural Resources‐Wildlife Resources Division (GADNR). Cedar Creek WMA consisted of upland loblolly pine stands, with interspersed areas of mixed pine‐hardwood forests, and expanses of hardwood dominated forests. In 2017, a turkey hunting season was open to the public from 25 March to 15 May, whereas in 2018 it spanned from 24 March to 15 May. We also collected data on the 4613‐ha B. F. Grant WMA (BFG) located in Putnam County, Georgia. The BFG was owned by the Warnell School of Forestry and Natural Resources at the University of Georgia and managed in partnership with the GADNR. The area consisted mostly of planted loblolly pine forests, hardwood forests, and agricultural fields used for cattle grazing and hay production. Turkey hunting season on BFG was split into three parts; the first was a youth‐only hunt which occurred from March 25 to April 2 in 2017 and March 24 to April 1 in 2018. The second hunt was an 80‐person quota from April 3 to April 9 in 2017 and April 2 to April 8 in 2018. The final hunt was open to the general public and occurred April 10–May 15 in 2017 and April 2–May 15 in 2018. For details on site‐specific conditions on BFG and CCWMA, see Wakefield et al. (2020).

### 2.2 Data collection and manipulation

We deployed ARUs (Song Meter Model SM2 and SM4, Wildlife Acoustics, Concord, MA, USA) to collect ambient sound from 1 March to 31 May. We deployed 10 ARUs on CWMA and 20 on SRS during 2014–2018, 15 on the Webb WMA Complex during 2015–2018, 16 on CCWMA in 2017, and 8 on BFG in 2017. We increased sampling efforts during 2018 in Georgia by deploying 20 additional ARUs on CCWMA and 10 on BFG. We placed ARUs >2 km apart to prevent multiple units from detecting the same call (Wakefield et al., 2020; Wightman et al., 2019). We attached ARUs to tree trunks approximately 3 m off the ground and placed an external microphone between 6 and 10 m above the ground on the same tree (Wightman et al., 2019). We placed ARUs at locations observed to have turkey activity based on field observations and global positioning system (GPS) locations of wild turkeys collected during previous research (Wightman et al., 2019). We used ambient sound recorded from 30 min prior to sunrise until 150‐min post‐sunrise as this is when >75% of vocalizations occurred (Wakefield et al., 2020, Wightman et al., 2019).

We used a convolutional neural network (CNN) developed to autonomously search for turkey gobbles (Wightman et al., 2022). We implemented the CNN in Python (Python Software Foundation, Wilmington, DE, USA) with the Keras library (Chollet, 2015) using a backend of the open‐source TensorFlow software developed by Google (Abadi et al., 2015). For each potential gobble selected by the CNN, a record was created containing call location in the spectrogram, date and time stamp, and a 3 s sound file of the potential gobble. We auditorily verified all selections and classified each as a true or false gobble then removed false positives, producing daily counts of gobbles on all sites. Due to time constraints associated with listening to all ambient sound recordings, we did not calculate false negatives; furthermore, false negatives are likely consistent across ARUs, sites, and years.

We collected weather data for SRS and CWMA from 2 weather stations located on SRS maintained by the U.S. Department of Energy and U.S. Department of Agriculture Forest Service. We used the most centrally located weather station on SRS to describe weather metrics associated with gobbling activity onsite. The second weather station was on the southern border of SRS, approximately 10.5 km from the center of CWMA and was used for gobbling evaluation on CWMA. For the Webb WMA Complex, CCWMA, and BFG, we collected weather metrics from the closest National Oceanic and Atmospheric Administration (NOAA) weather station. The closest weather station to the Webb WMA Complex was located in Varnville, SC (35 km), whereas the closest station to CCWMA (25 km) and BFG (35 km) was near Eatonton, GA. Although previous authors have suggested the potential for placing weather stations at each ARU (Palumbo et al., 2019; Wightman et al., 2019), such a study design was not logistically feasible. We offer that using weather data collected on the same study site or within the distances detailed above is sufficient for detailing how daily changes in local weather conditions influence gobbling activity. We calculated mean daily values from 15‐min weather recordings from 30 min prior to 150 min after sunrise for temperature (C°), relative humidity percentage, and wind speed (kph). For barometric pressure (mb), we calculated the mean for each morning and then subtracted it from the prior morning to get a change in barometric pressure. For precipitation, we classified whether rain occurred (Yes = 1, No = 0) from 30 min before to 150 min after sunrise.

### 2.3 Data analysis

Our final dataset included time series data for all weather variables (scaled by subtracting variable means from observed values and dividing by the standard deviation) and daily gobbling counts. With the spatially and temporally coupled data, we used state‐space modeling to evaluate the effects of weather variables on daily gobbling activity. The state‐space model accounted for correlated observations and included observation error while modeling the influences of weather variables on gobbling activity. We used a hierarchical state‐space model that allowed us to decompose temporally correlated weather data and gobbling counts into a process variation and observation error (Kery & Schaub, 2012). With the weather variables being the parameters of interest, the state‐space model allowed us to investigate the process variation in gobbling counts relative to stochasticity in the weather variables. We calculated Pearson's correlation coefficients to test for collinearity between each of our covariates and excluded covariates with a *r* > .60. We fit the state‐space model within the jagsUI package (Kellner, 2018) in program R (R Core Team, 2020) to estimate the effects of weather on daily gobbling activity.

We fit the Bayesian state‐space model to counts of daily gobbles (N) at each site (K) during each year (i). We treated daily gobbling counts like counts in a population model but we modeled the abundance of gobbles instead of animals. The process model was:

$$\begin{array}{c} r_{\text{expected (t)}}={\text{X}}_{\log\;\text{(N}\text{[t−1]}\text{,k,i)}}+{\text{Site}}_{\text{(k)}}+{\text{β}}_{\text{temperature}}*{\text{X}}_{\text{temperature (t,k,i)}}+{\text{β}}_{\text{wind}}*{\text{X}}_{\text{wind (t,k,i)}}\\ \;+{\text{β}}_{\mathrm{bp}}*({\text{X}}_{\text{bp (t,k,i)}}-{\text{X}}_{\text{bp (t−1,k,i)}}+{\text{β}}_{\text{humidity}}*{\text{X}}_{\text{humidity (t,k,i)}}+{\text{β}}_{\text{precipitation}}*{\text{X}}_{\text{precipitation (t,k,i)}}\\ \;\text{+ Year + log (Units).} \end{array}$$

$$r_{t}\sim \text{Normal}\;\left(r_{\text{expected [t]}},\tau_{\text{process}}\right).$$

$$\mathrm{Log}\left(N_{t+1}\right)=\log\left(N_{t}\right)+r_{t}$$

Where *r*_*expected(t)*_ was the expected change in daily gobbling activity, Site was the fixed effect for each of the 5 sites, β_temperature_ was the coefficient for the effect of temperature in matrix X_temperature_, β_wind_ was the coefficient for the effect of wind in matrix X_wind_, β_bp_ was the coefficient for the effect of the change in barometric pressure in matrix X_bp_, β_humidity_ was the coefficient for the effect of humidity in matrix X_humidity_, β_precipitation_ was the coefficient for the effect of precipitation in matrix X_precipitation_, Year was modeled as a random effect, and Units was an offset term used to account for the number of ARUs recording. We modeled the observation process as follows: *y*_*t,k,i*_ *~ Poisson(log(N*_*t*_ *))* where *y*_*t,k,i*_ was the logged observed number of gobbles each day(t) at each site during each year. We calculated 95% credible intervals for each parameter estimate of interest. For the random effect of year and to account for process variation, we used a gamma distribution for the priors with a precision of 0.001. For the rest of the parameters, we used a normal distribution with a mean of 0 and a precision of 0.001. We used Markov chain Monte Carlo (MCMC) to estimate the posterior distributions of the model parameters. We generated 3 MCMC chains using a thinning rate of 10,000 iterations per chain and 2500 burn‐in values. To check for convergence, we investigated trace plots of the MCMC chains and used Gelman‐Rubin statistic to calculate *R*‐values, with *R*‐values < 1.1 indicating model convergence (Gelman et al., 2004).

## 3 RESULTS

We autonomously searched 75,858 h of ambient sound for potential gobbles. The CNN identified 324,236 potential gobbles of which 194,655 (60%) were true gobbles (Table 1). Mean gobbles per ARU from 1 March to 31 May were highest on SRS (937 ± 326, mean ± SD), 11% less on CWMA (838 ± 404), 52% less on the Webb WMA complex (443 ± 120), 46% less on BFG (507 ± 38), and 61% less on CCWMA (369 ± 130, Table 1).

**TABLE 1:** Detections, gobbles, and gobbles per autonomous recording unit (ARU) for Crackerneck Wildlife Management Area (CWMA), Savannah River Site (SRS), and the Webb Wildlife Management Area Complex (Webb) in South Carolina and Cedar Creek Wildlife Management Area (CCWMA) and B.F. Grant Wildlife Management Area (BFG) in Georgia from 2014 through 2018

Site	Year	Detections	Gobbles	Gobbles (%)	Gobbles per ARU
CWMA	2014	19,214	14,242	74	1424
CWMA	2015	10,614	6234	59	623.40
CWMA	2016	12,458	7032	56	703.20
CWMA	2017	14,941	10,518	70	1051.80
CWMA	2018	8246	3892	47	389.20
SRS	2014	29,138	21,484	74	1074.20
SRS	2015	22,409	17,242	77	862.10
SRS	2016	25,039	18,236	73	911.80
SRS	2017	35,043	27,366	78	1368.30
SRS	2018	16,434	9454	58	472.70
Webb	2015	12,476	8063	65	537.53
Webb	2016	12,946	8305	64	553.67
Webb	2017	9096	4701	52	313.40
Webb	2018	11,793	5524	47	368.27
CCWMA	2017	5176	4437	86	277.31
CCWMA	2018	37,795	16,606	44	461.28
BFG	2017	15,014	3839	26	479.88
BFG	2018	26,404	7480	29	534.29

**FIGURE 2:**
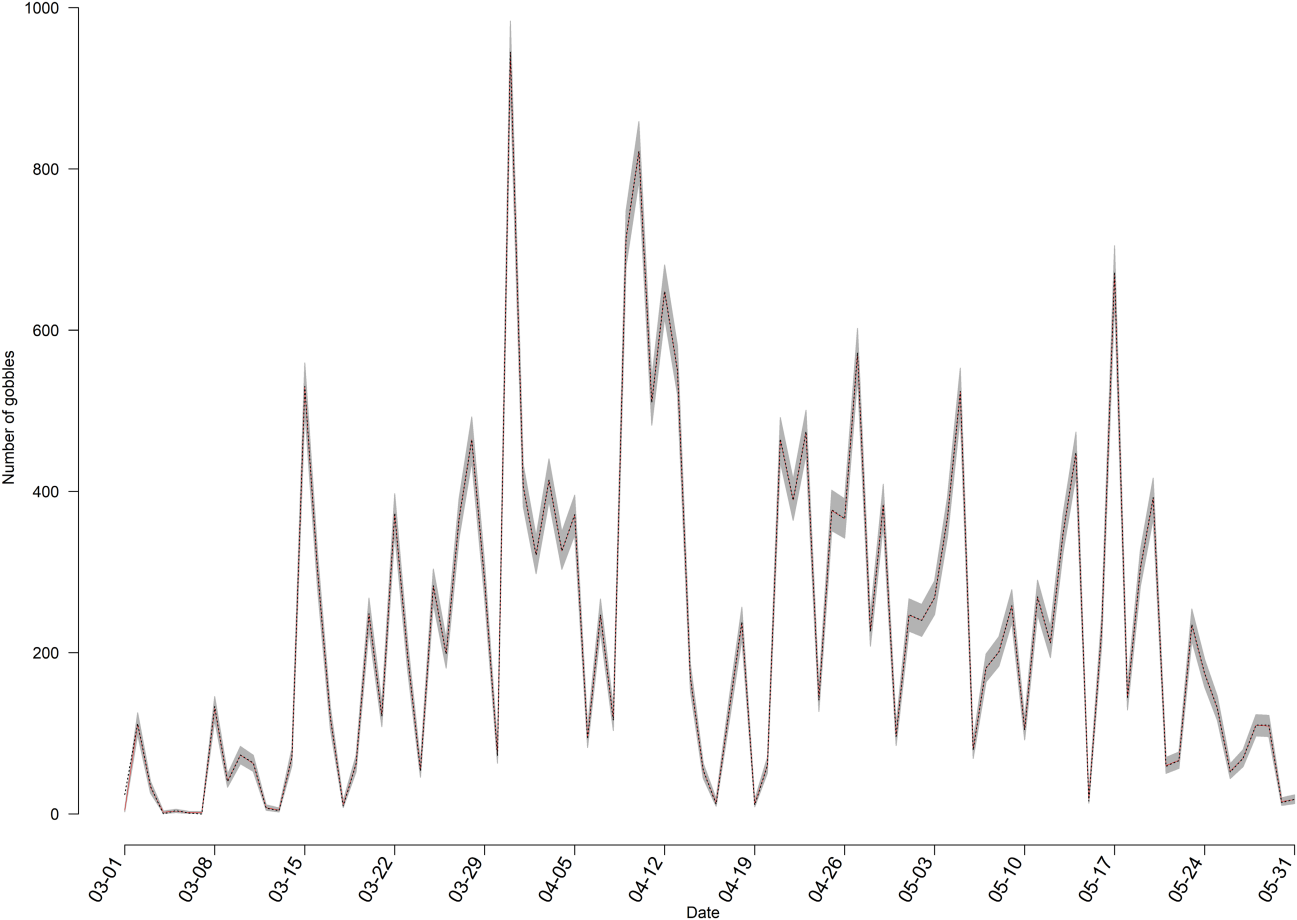
Predicted daily gobbling activity from state‐space model (dotted line) with 95% credible intervals (shaded gray) compared with observed daily gobbling activity (red line) on the Savana River Site in South Carolina, USA, 2014

**FIGURE 3:**
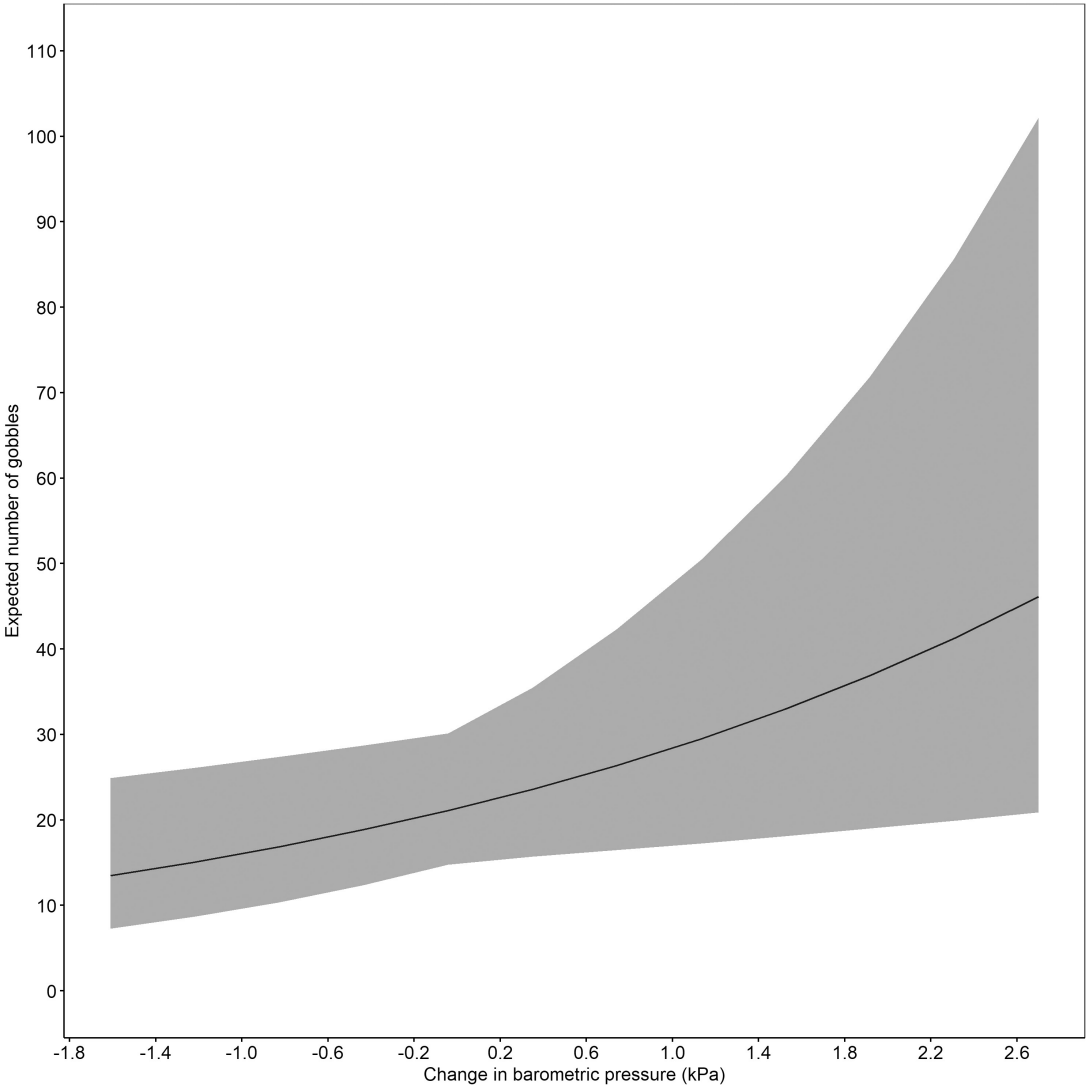
Expected number of gobbles (*r*_expected(t)_*20) with 95% credible intervals as a function of change in barometric pressure (mb) across all 5 sites in South Carolina and Georgia, USA, 2014–2018

**FIGURE 4:**
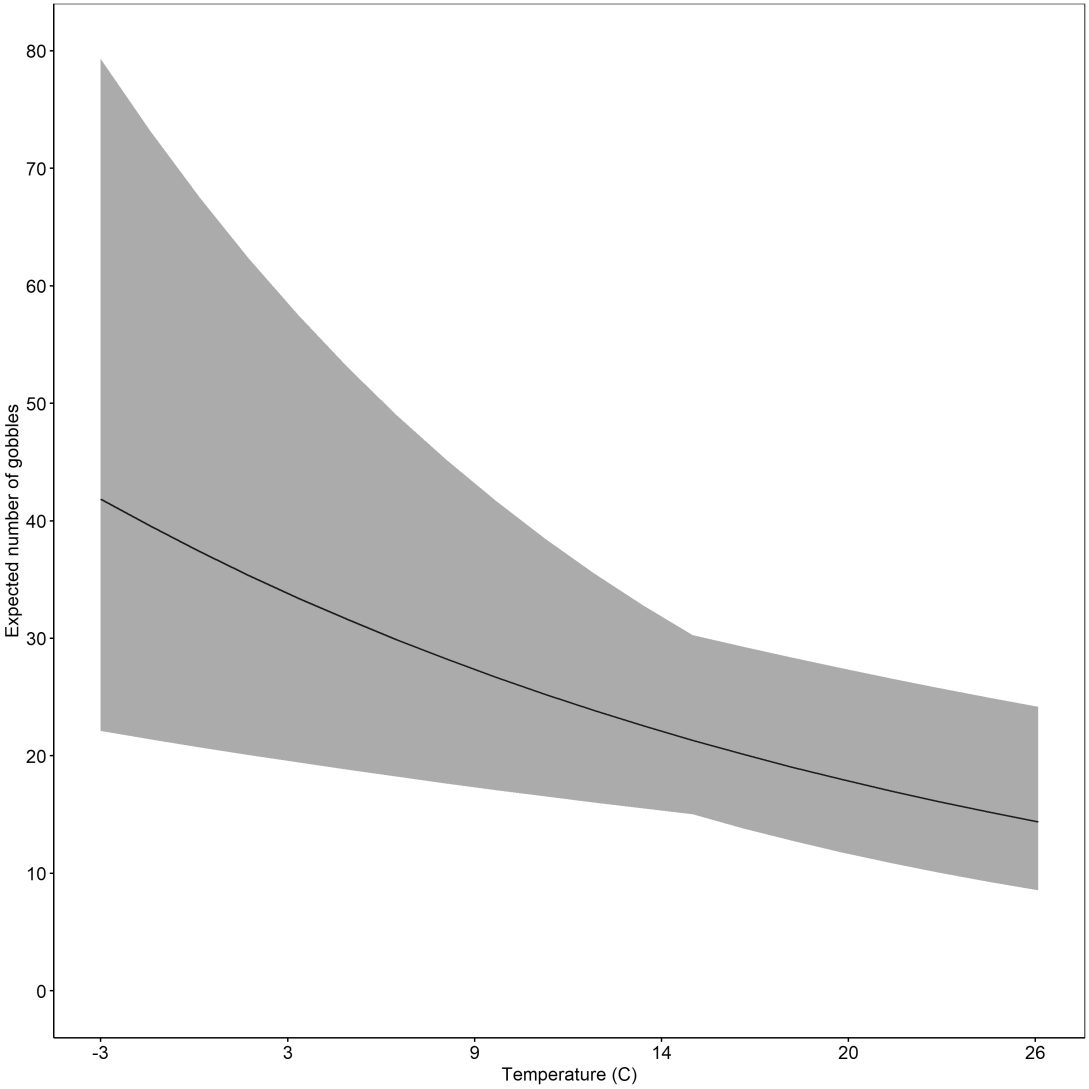
Expected number of gobbles (*r*_expected(t)_*20) with 95% credible intervals as a function of temperature (°C) across all 5 sites in South Carolina and Georgia, USA, 2014–2018

**FIGURE 5:**
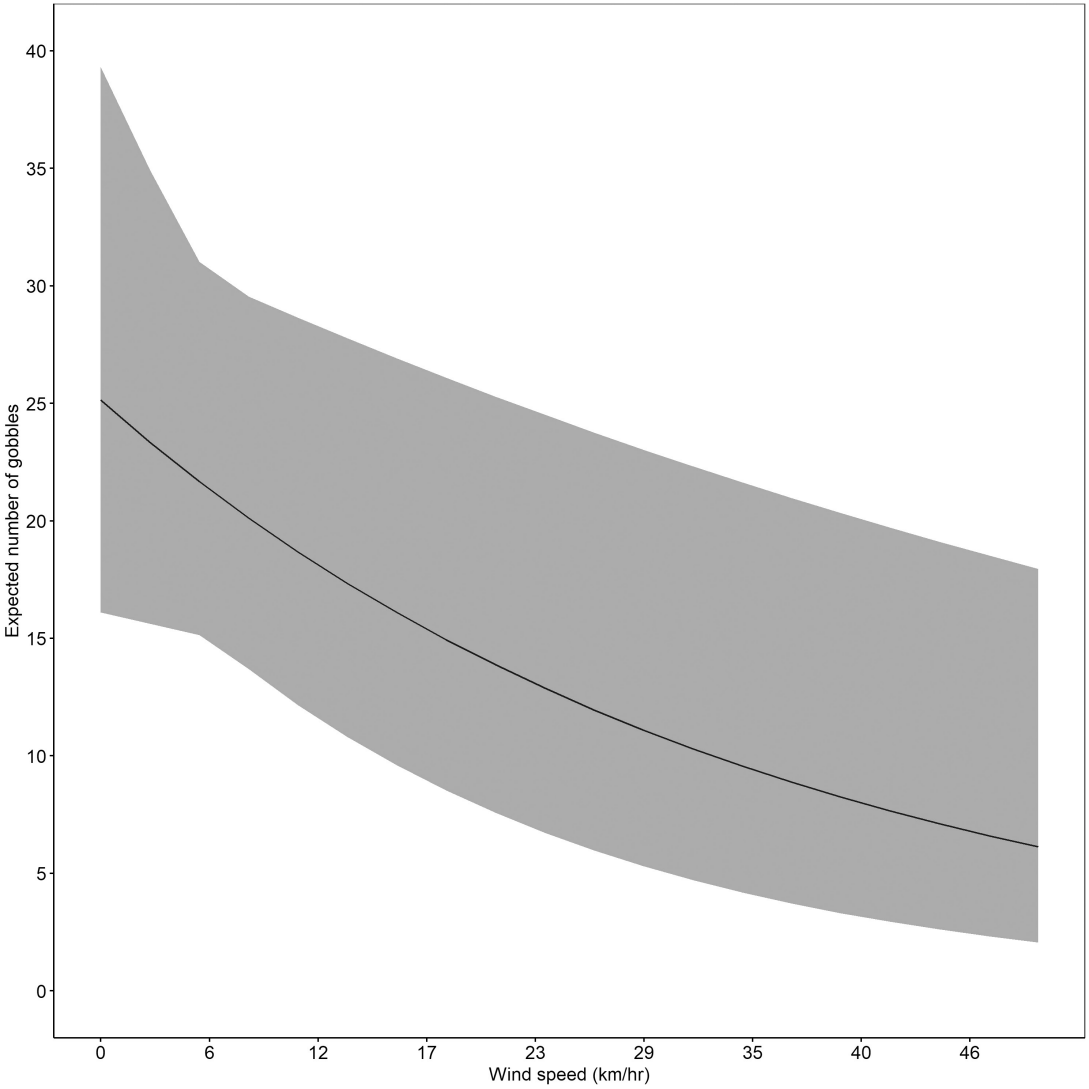
Expected number of gobbles (*r*_expected(t)_*20) with 95% credible intervals as a function of wind speed (km/hr) across all 5 sites in South Carolina and Georgia, USA, 2014–2018

The state‐space model accurately predicted gobbling activity compared with our observed gobbling activity (Figure 2), and *R*‐values indicated model convergence (Table 2). Results from the state‐space model indicated the occurrence of rain most impacted (negatively) gobbling activity (Table 2). Where the mean expected number of daily gobbles would be 21 (CrI = 15, 30) without rain, compared to 12 (CrI = 7, 22) if rain occurred. Conversely, an increase in barometric pressure from one day to the next was positively associated with gobbling activity (Figure 3, Table 2). We found gobbling activity was negatively influenced by increased temperatures (Figure 4, Table 2) and by greater wind speed with the largest effect occurring when wind speeds exceeded 10 kilometers per hour (Figure 5, Table 2). Humidity had no effect on the average predicted rate of change in gobbles across all study sites and years (Table 2).

**TABLE 2:** Parameters and associated means, standard deviations (SD), and credible intervals from a state‐space model evaluating the relationship between daily gobbling activity by male wild turkeys and weather variables for Crackerneck Wildlife Management Area (CWMA), Savannah River Site (SRS), and the Webb Wildlife Management Area Complex (Webb), in South Carolina and Cedar Creek Wildlife Management Area (CCWMA) and B.F. Grant Wildlife Management Area (BFG) in Georgia from 2014 through 2018

Parameters	Mean	SD	2.50%	97.50%	*R*‐value
CWMA	0.20	0.115	−0.01	0.42	1
SRS	0.11	0.09	−0.04	0.29	1
Webb	0.10	0.12	−0.13	0.33	1
CCWMA	−0.01	0.15	−0.36	0.23	1
BFG	−0.07	0.15	−0.31	0.26	1
Temperature	−0.21	0.05	−0.30	−0.11	1
Wind	−0.16	0.05	−0.33	−0.13	1
Barometric Pressure	0.28	0.09	0.14	0.48	1
Humidity	0.09	0.06	−0.02	0.21	1
Precipitation	−0.56	0.12	−0.76	−0.29	1
2014	0.01	0.07	−0.13	0.18	1
2015	0.02	0.07	−0.11	0.19	1
2016	0.02	0.07	−0.11	0.18	1
2017	−0.01	0.07	−0.19	0.09	1
2018	−0.03	0.07	−0.20	0.09	1

## 4 DISCUSSION

Previous literature detailing how weather influences gobbling activity has reported contradictory results (Bevill, 1973; Kienzler et al., 1996; Miller et al., 1997a; Palumbo et al., 2019; Scott & Boeker, 1972), leading to uncertainty in the relative contribution of weather variables to daily fluctuations in gobbling activity. We used the most comprehensive dataset currently available on wild turkey gobbling activity, coupled with local weather metrics, to evaluate relationships between gobbling activity and weather. Collectively, our findings suggest weather variables can influence daily gobbling activity and are at least partially responsible for oscillations in gobbling activity throughout the spring reproductive season.

Gobbling is a behavior males use to attract females and ensure reproductive opportunities (Buchholz, 1997). However, gobbling increases predation risk as predators are attracted to calls, so males must balance increasing predation risk with attracting mates (Burk, 1982; Jennions & Petrie, 1997; Tuttle & Ryan, 1981). In birds, weather conditions can also increase predation risk; therefore, males may adopt varying calling strategies in response to weather conditions (Carr & Lima, 2010; Digby et al., 2014). We found rain had the greatest influence on gobbling activity, as has been shown in earlier works (Bevill, 1973; Kienzler et al., 1996). During rain events, calling males may be more vulnerable to predation as their hearing and vision, which they rely on for detecting predators, are compromised (Candolin & Voigt, 1998; Healy, 1992; Hedrick, 2000). Furthermore, during rain events sound attenuation is increased, making it harder for the gobble to be heard by other individuals (Lengagne & Slater, 2002). Alternatively, rain may simply reduce the ability of the ARU to detect gobbles, although we detected 21,180 gobbles during rain events and literature on other bird species reported that rain negatively influenced calling (Bruni et al., 2014; Digby et al., 2014; Staicer et al., 1996). We posit that the influence of rain on gobbling activity recorded by ARUs is likely a combination of detection and ecology, but when reporting gobbling chronology should be considered.

Increases in animal activity and calling have previously been associated with increases in barometric pressure across multiple species (Oseen & Wassersug, 2002; Pellegrino et al., 2013; Wellendorf et al., 2004; Zagvazdina et al., 2015). Changing barometric pressure is a well‐known predictor of storm fronts, with barometric pressure falling as inclement weather approaches and rising as the storm system dissipates (Breuner et al., 2013; Saucier, 2003). Miller et al. (1997a) found no relationship between gobbling and barometric pressure, but we observed an increase in barometric pressure from one day to the next resulted in increased gobbling activity. Given changes in barometric pressure and its relationship to inclement weather such as rain, we conclude that this relationship is best explained by turkeys gobbling more in weather conditions not associated with storm systems. Furthermore, if storms represent a period of reduced signal efficacy, it is plausible that turkeys are increasing gobbling activity as storm fronts pass to compensate for lost signaling time.

Extant literature has noted a significant relationship between decreased calling and higher temperature in various birds that use auditory courtship behaviors (Gudka et al., 2019; Hansen & Guthery, 2001). Vocalization and thermal relationships are likely related to overheating and higher metabolic rates that can occur with increased ambient temperatures, especially for endotherms who use energetically costly courtship behaviors (Dillon et al., 2010; Silva et al., 2015). We observed a similar relationship between higher temperatures and gobbling activity but note that previous studies at southern latitudes reported no relationship between temperature and gobbling activity (Miller et al., 1997a; Palumbo et al., 2019), whereas at more northern latitudes studies have reported positive relationships between gobbling and temperature (Kienzler et al., 1996). Male wild turkeys are primarily hunted during the spring reproductive season, and spring harvest is the primary cause of mortality for males (Chamberlain et al., 2012). One could speculate that reduced gobbling activity during warmer temperatures could be related to the removal of males causing drops in gobbling later in the sampling period when temperatures are warmer. However, given that we had 5 years of data on an un‐hunted site where gobbling continued until the end of the sampling period (Figure 2), we suspect that this is not the case. Wild turkeys at southern latitudes may reduce gobbling at higher temperatures, but we offer that the pattern may not be similar at northern latitudes.

Sound attenuation increases at greater wind speeds, and previous studies have demonstrated wind can negatively influence calling frequency and the ability to hear calls in multiple species (Lengagne et al., 1999; Lengagne & Slater, 2002; Yip et al., 2017). We observed that greater wind speeds had a negative effect on daily gobbling, consistent with previous studies (Bevill, 1973; Kienzler et al., 1996; Miller et al., 1997a). The ability for either human observers or ARUs to detect gobbling as wind speeds increase may be diminished (Kienzler et al., 1996). Alternatively, during high wind speeds birds may change behaviors as perceived risk increases, as individuals have increased difficulty detecting predators due to confusion with moving vegetation (Boyko et al., 2004; Carr & Lima, 2010). We suspect males may be less inclined to gobble as wind speeds increase because the desired outcome from calling may be limited by the ability of receptive females to hear the call and because predation risk may increase.

Wakefield et al. (2020) used the same modeling approach that we used, focused on describing the influences of female reproduction (laying or incubating) and cumulative removal of males on daily gobbling activity. Wakefield et al. (2020) found the proportion of females in reproduction positively influenced gobbling activity, but the impact of male removal at the same time had a greater negative impact on gobbling activity. We also recognize other variables not measured in our or previous studies may be contributing to variation in daily gobbling, such as varying levels of testosterone in males, interactions/encounters with females, and population vital rates such as male age structure (Chamberlain et al., 2018; Miller et al., 1997a; Wakefield et al., 2020).

As gobbling activity is positively correlated with hunter satisfaction and linked to reproduction, it is often a key determinant used by state agencies when considering regulatory frameworks (Bevill, 1975; Casalena et al., 2011; Hoffman, 1990; Isabelle & Reitz, 2015; Little et al., 2001). Given our results, we suggest that when describing gobbling activity, managers should account for how weather patterns may influence gobbling chronology. Weather variables should be coupled with site‐specific reproductive timing and harvest data to fully understand gobbling chronology on a given site. We recommend future studies investigate the relationship between daily gobbling activity, weather, and reproductive phenology of females in an un‐hunted population and populations subjected to varying hunting seasons and harvest rates.

## AUTHOR CONTRIBUTIONS

**Patrick H. Wightman:** Conceptualization (equal); data curation (lead); formal analysis (lead); writing – original draft (lead). **James A. Martin:** Conceptualization (supporting); formal analysis (supporting); writing – review and editing (supporting). **John C. Kilgo:** Data curation (supporting); funding acquisition (supporting); project administration (supporting); writing – review and editing (supporting). **Emily Rushton:** Funding acquisition (equal); project administration (supporting); writing – review and editing (supporting). **Bret A. Collier:** Conceptualization (supporting); data curation (supporting); funding acquisition (equal); project administration (equal); writing – review and editing (supporting). **Michael J. Chamberlain:** Conceptualization (equal); data curation (supporting); funding acquisition (equal); project administration (lead); writing – review and editing (lead).

## CONFLICT OF INTEREST

None.

## Data Availability

All raw gobbling and weather data from this study can be accessed on Dryad (DOI https://doi.org/10.5061/dryad.573n5tb9k).
